# Regularizing daily routines for mental health during and after the COVID-19 pandemic

**DOI:** 10.7189/jogh.10.020315

**Published:** 2020-12

**Authors:** Wai Kai Hou, Francisco TT Lai, Menachem Ben-Ezra, Robin Goodwin

**Affiliations:** 1 Department of Psychology, Centre for Psychosocial Health, The Education University of Hong Kong, Hong Kong SAR, China; 2 Jockey Club School of Public Health and Primary Care, The Chinese University of Hong Kong, Hong Kong SAR, China; 3 School of Social Work, Ariel University, Ariel, Israel; 4 Department of Psychology, University of Warwick, Coventry, UK

The SARS-Cov-2 (COVID-19) pandemic, though far from concluded, has already had an enormous psychological [1] and economic [2] impact on over half of the world’s countries and regions. Everyday life can actually be seen as the fundamental context for resilience during trauma and chronic stress [3]. In response to the pandemic, different forms of lockdown, quarantine, and social/physical distancing have restricted interactions both within and across regions and countries. This has threatened basic livelihoods and mobility, reduced interpersonal interactions, and led to new workforce patterns and the suspension of schools and higher education. These changes in major life domains resemble the functional impairment consequential to mental disorders such as depression and place a large number of people at greater risk of poor mental health. In the latest published guidelines on mental health and psychological aspects of COVID-19, the World Health Organization (WHO) [4] suggests regular activities for children confined at home and new routines for vulnerable adults, including those who are older and /or with chronic health conditions. This advice is reflected in advice by Governments in the UK (PHE), the US (CDC), and elsewhere.

Regularized routines, such as those offered above, can buffer the adverse impact of stress exposure on mental health [5,6], but it is unclear how this message can best be conveyed. Below we suggest a number of options and important caveats to maximize the efficacy of this advice, and its positive impact on mental health. The guidelines will be useful for enhancing population mental health during and after this pandemic and, arguably, other epidemics in the future.

First, it is important to recognize that daily routines are likely to differ in their impact on mental health. Daily routines can be parsed into two types [7]. *Primary routines* are behaviors necessary for maintaining livelihood and biological needs, such as hygiene, sleep, and eating. *Secondary routines* reflect individual circumstances, motivations and preferences, and include exercising, leisure/social activities, and practices associated with work or study, including keeping oneself on time and meeting goals and targets. During a pandemic, some routines are disrupted as a result of stress (eg, sleep) while other disruptions result from economic factors (eg, work activities). Routines are often terminated due to other contextual restrictions, for example, face-to-face interactions with relatives, friends, or coworkers. With our multitude of daily activities, disruption and termination can often co-occur. Because primary routines regularize the overall structure of daily living, disruption and termination of primary routines have a more pivotal role in mental health during acute stress [3,5,6].

Second, primary and secondary daily routines can be usefully consolidated and replaced, while new routines can be added [8]. Consolidation of existing routines may mean, for example, that time at home is used for household tasks or indoor leisure activities. Replacement could include using telephone/video calls or social media instead of face-to-face interaction. Adaptive new routines can be added to complete the everyday life structure, for example, by spending more time exercising or ensuring personal and household hygiene. During a pandemic, new routines might include lengthier handwashing (perhaps to a song) or other preventive measures such as wearing a mask and washing hands more often. These behaviors restore a sense of normalcy, controllability, and predictability. It is important to note that some of them, for example the regular use of mask for infection prevention, will vary across sociocultural contexts.

Two principles guide the sustainment of daily routines. Primary routines (eg, regular healthy diet, sleep, and personal hygiene) should be prioritized over secondary routines including leisure and social activities, exercising, and work/study in order to maintain an overall regular daily living that directly enables positive mental health. Consolidation should be prioritized prior to replacement and addition, because fewer resources are needed for consolidating disrupted routines relative to replacing or adding new ones. During times of high stress, consolidation of existing social ties with family and friends is preferred over the addition of new social partners [9].

Prompt dissemination of these guidelines could be implemented through traditional broadcast media (print, radio, and television) and digital media including websites and social media platforms. These guidelines can further be strengthened face-to-face by a national team of trained community health workers who are young, less susceptible to the COVID-19, and in need of employment opportunities due to the economic downturn [10]. Alternatively, nationwide voluntary schemes such as the UK’s GoodSam scheme provide useful personnel. These guidelines could also be strengthened as part of a health promotion strategy for disadvantaged groups such as older adults living alone and low-wage essential workers (eg, cleaners, couriers, grocery workers) who, through their continuous interactions with a range of others, are more vulnerable to infection.

**Figure:**
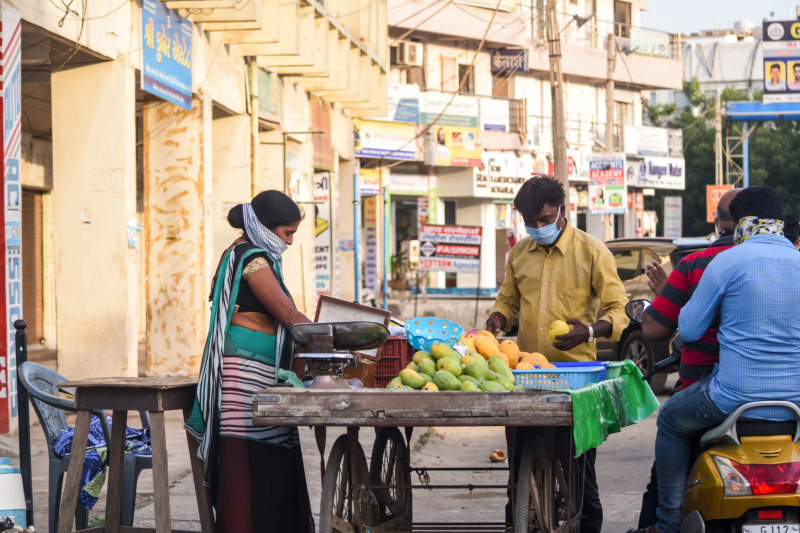
Photo: From unsplash.com.

Unprecedented by any previous public health crises, the COVID-19 pandemic puts a pause on people’s hectic daily living. Interventions are needed to direct focus towards the role of daily living in order to promote psychological resilience. Mass education and non-specialist care can be fruitfully recalibrated to emphasize the sustainment of regular daily routines to ensure positive mental health during and after a pandemic.
